# A pilot study: a possible implication of *Candida* as an etiologically endogenous pathogen for oral lichen planus

**DOI:** 10.1186/s12903-020-1042-8

**Published:** 2020-03-14

**Authors:** Hong He, Xinyu Xia, Haiping Yang, Qiao Peng, Jiaoer Zheng

**Affiliations:** 1 The Affiliated Stomatology Hospital, Zhejiang University School of Medicine, Yan’an Road, Hangzhou, 310006 China; 2 Key Laboratory of Oral Biomedical Research of Zhejiang Province, Hangzhou, China; 3 School of Medicine, Zhejiang University, Hangzhou, China

**Keywords:** Oral lichen planus, Etiology, Endogenous infection, *Candida albicans*, ITS sequence, Homology similarity coefficient S_AB_

## Abstract

**Background:**

The aim of this study was to investigate the prevalence and genotypic profiles of *Candida albicans* in patients with oral lichen planus (OLP).

**Materials and methods:**

Positive rates and genotypic profiles of *Candida albicans* strains from OLP patients and healthy controls were analyzed. Random amplified polymorphic DNA and internal transcribed spacer of ribosome DNA polymerase chain reactions were used to sequence the DNA of these strains, and then their genetic similarity was measured using BLAST, UIV Band, and Vector NTI Suite Sequence Analyses Software.

**Results:**

The prevalence of *C. albicans* strains detected from erosive-OLP, non-erosive OLP, and normal individuals was 18.87, 18.75, and 7.92%, respectively. Four different genotypes were revealed by the two methods. To be specific, type I was found only in the healthy subjects; type II a and II b were found in non-erosive OLP, and type III was identified in erosive OLP. Intragroup similarity coefficients, i.e. S_AB_ were 100%, and inter-groups similarity coefficients, i.e. S_AB_ were less than 30%.

**Conclusions:**

The genotypic results of *C. albicans* in OLP revealed an endogenous rather than exogenous infection of *C. albicans.* In addition*,* a possible pathogenic role of *C. albicans* in OLP, with the etiologic sense contributing to a more proper recognition on the pathogenesis, development, and progression of OLP, as well as some strategies for its diagnosis and treatment were identified.

## Background

Oral lichen planus(OLP)is a common chronic inflammatory oral mucosal disease affecting 1–2% of the general population [1–3]. It is considered as a potentially malignant disorder with a deteriorated transformation prevalence of 0–1% according to the World Health Organization [4, 5]. While the etiology of OLP remains to be elucidated, the existing evidence suggests that microbial infection, psychological disorders, allergies, and immunodeficiency are closely associated with the pathogenesis of OLP [5–7].

Considering the oral microorganisms, *Candida* species are the commensal fungus in the mucosal flora of healthy individuals, which also have a crucial role in OLP [8–10]. Among *Candida* species*, Candida albicans* (*C. albicans*) is the predominant specie in the OLP patients [11–13]. Arora et al. have investigated the prevalence and phenotypic variation of *Candida* species in OLP cases, and found that *C. albicans* constituted the majority of the five identified *Candida* species [12]. Thus, *C. albicans* from OLP has become a popular research topic over recent years in many clinical and laboratory studies.

Initially, the researches were focused on the phenotypic presence of *C. albicans* in OLP; however, due to the low resolution of phenotypic analysis they failed to answer the following basic questions: whether the pathogen is a fungus; whether *C. albicans* is an initial pathogen or a symbiont; whether it facilitate the infection in OLP, or vice versa; and whether *C. albicans* is a probable antigen for OLP. It is worth noting that a close correlation might exist between genotypic profiles and virulence attributes [14]. Moreover, genotyping can provide information about DNA and has stronger resolution than phenotyping necessary to distinguish variants among individuals or stages [14, 15]. Therefore, the relationship between genotypes of *C. albicans* and OLP needs to be further investigated.

In this study, genotyping analyses using BLAST, UIV Band, and Vector NTI Suite Sequence Analysis software were conducted to investigate the relationship between OLP and genotypes of *C. albicans*. Combination of random amplified polymorphic DNA **(**RAPD) and internal transcribed spacer (ITS) [10, 16, 17] of ribosome DNA polymerase chain reaction (PCR) was used to intergroup genotype *C. albicans* isolates from healthy individuals and OLP patients. The results showed that the phenotypes and the intragroup genotype of *C. albicans* were homologous; while they were genotypically heterogeneous among erosive (E)-OLP, non-erosive (NE)-OLP and healthy individuals. Both methods revealed three discrepant subtypes of *C. albicans* in clinical different stages of OLP, and one different subtype in healthy controls. Thus, we assumed that endogenous infection rather than exogenous infection of *C. albicans* might be the vital factor in OLP etiology.

## Materials and methods

This study was approved by the local research ethics committees, the ethics committee of the Affiliated Second Hospital, and the ethics committee of the Affiliated Stomatology Hospital, Zhejiang University School of Medicine, China. All patients provided written informed consent. The protocols were reviewed and approved by the local institutional review boards (IRB). The IRB numbers for our medical ethic files were No. 2015 (17) of the stomatology hospital and 2010 (137) of the second hospital both affiliated to School of Medicine Zhejiang University. All participants underwent training session to minimize the undesirable discrepancy.

**Fig. 1:**
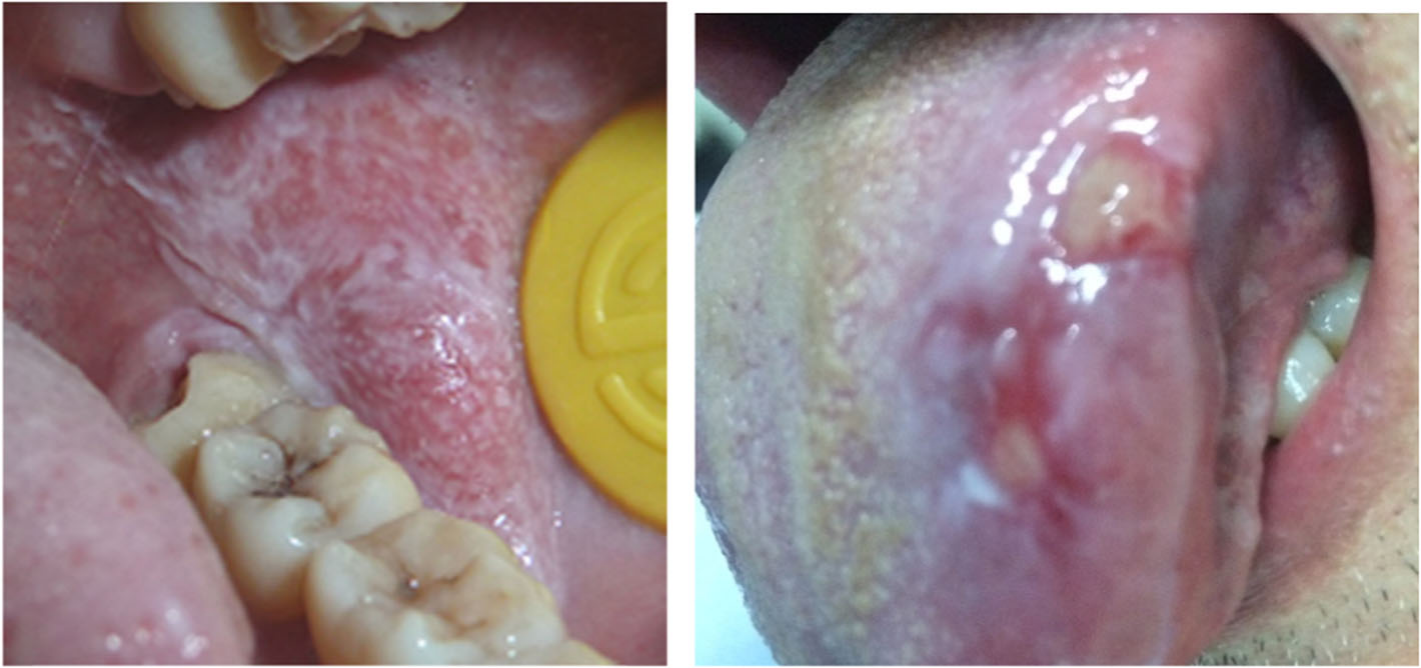
OLP Lesions in left buccal (a non-erosive one) and left tongue (an erosive one)

G*power software was used for power calculation; a power was 94% with α = 0.05 for the standard deviation in groups of patients and control. A total of 250 subjects as sample size (aged between 16 and 86 years), including 149 patients with OLP and 101 age-sex-matched healthy volunteers (*P* > 0.05), were recruited at the Affiliated Hospitals of Zhejiang University between February 2010 and March 2016 (Table 1 and Fig. 1). The patients were diagnosed with OLP based on clinical and histopathological criteria, as well as the criteria of Chinese Stomatological Association of Oral Medicine. The 101 healthy individuals had no oral mucosa diseases. The exclusion criteria were as follows: patients having full or partial dentures, those taking broad-spectrum antibiotics, antifungals, glucocorticoids, or immunosuppressive agents over a long period of time or within last 3 months, and those with systemic diseases, including diabetes, thyroid hypofunction, and immune deficiency.

**Table 1:** Clinical features and positive culture rate of *Candida albicans* strains of the subjects(^**^P = 0.024, ^*^P = 0.044)

Groups	Gender		Age	Positive cases	Positive rate
	M	F	(Mean)		
NE-OLP(*n* = 96)	47	49	48.22 ± 16.43	18	18.75%^**^
E-OLP(*n* = 53)	27	26	51.37 ± 15.78	10	18.87%^*^
Controls(*n* = 101)	51	50	49.69 ± 17.19	8	7.92%

### Isolates

From experience, the authors have thought that swabbing is better than rinse to isolate *Candida* species from the oral cavity. All the sample swabs were taken from subjects’ oral mucosal membrane. Lesion regions in patients with OLP and eight regions in the healthy individuals’ oral mucosa were consistently swabbed. The eight regions in healthy individuals’ oral mucosa were bilateral buccal, palate, back and two sides of abdominal tongue, and vestibules. The team members were trained for standard performance actions. The clinical isolates were randomly labeled, and researchers who conducted phenotyping and genotyping analyses were blinded to all information of the isolates. All isolates were first analyzed using conventional microbiological identification methods [18, 19], and those with the morphological characteristics of *C. albicans* were analyzed using germ tube test and the API 20C AUX identification kit (Analytical Profile Index; BioMe’rieux S.A., France).

### DNA extraction

DNA was extracted using the Biospin Fungus Genomic DNA Extraction Kit (Bioflux, China) following the manufacturer’s protocol. DNA concentration was examined using the Ultraviolet Spectrophotometer (Pharmacia Biotech, USA, 07450).

### RAPD fingerprinting and S_AB_ analysis

RAPD was conducted as described [20]. Two primers (C1: 5′-ACGGTACACT-3′; C2: 5′-GTTCCGCCC-3′) were used in this study. After amplification, the products were electrophoresed in 2% of agarose gel. After staining with ethidium bromide (Kaiser Germany), the PCR products were qualified using the UIV Band system. Information about the location and relative molecular mass of the main amplified bands was extracted for calculating the genetic similarity coefficient S_AB_ between any two bands. Strains were categorized into several families that share gene homology at a criterion of S_AB_ = 0.8.

### ITS sequence determination

ITS analysis was conducted as described in a previous study (White et al.). PCR amplification was performed with primers (5′-GGAAGTAAAAGTCGTAACAAGG-3′; 5′-GCTGCGTTCTTCATCGATGC-3′) that were designed based on the conserved regions of ITS 1 and ITS 2 rRNA genes. The amplified DNA fragment included the intervening 5.8S gene and the ITS 1 and ITS 2 noncoding regions. Primers were synthesized by the Eppley Molecular Biology Core Laboratory (NE, USA). The amplified PCR products were purified and sequenced by TaKaRa Bio-Engineering Co., Ltd. (Dalian, China). BLAST was used to compare the DNA sequence with standard ITS sequences of *C. albicans* in the GenBank at National Center for Biotechnology Information to determine the taxonomy of the isolates. Intra-species sequence similarity and variation for isolates were determined using the Vector NTI Suite software (www. liax.cn) and visually confirmed using pairwise nucleotide alignments. Referenced isolates were also aligned. The similarities of the sequences were determined with the expectation frequency minimized to 0.0001.

### Statistical analysis

A *P* value < 0.05 was set as the standard statistical significance. All calculations were performed by χ^2^ test using SPSS statistical software (SPSS 25.0; SPSS Inc., Chicago, IL, USA).

## Results

### Positive rate of *C. albicans* culture—prevalence of *C. albicans* in OLP

The results showed that *C. albicans* were identified from 16 of 96 NE-OLP (16.67%), 10 of 53 E-OLP (18.87%), and 8 of 101 healthy controls (7.92%) (Table 1)*.* The prevalence of *C. albicans* in NE-OLP and E-OLP were significantly higher than that in the healthy symbiotic group (χ^2^ test, *P* < 0.05 respectively as 0.024 and 0.044), but no significant difference was found between NE-OLP and E-OLP (χ^2^ test, *P* > 0.05) (Table 1). Using germ tube test and the API 20C AUX identification kit, eight isolates of *C. albicans* from NE-OLP, two from E-OLP, and two from the healthy controls were identified.

### RAPD and UIV band analysis

**Fig. 2:**
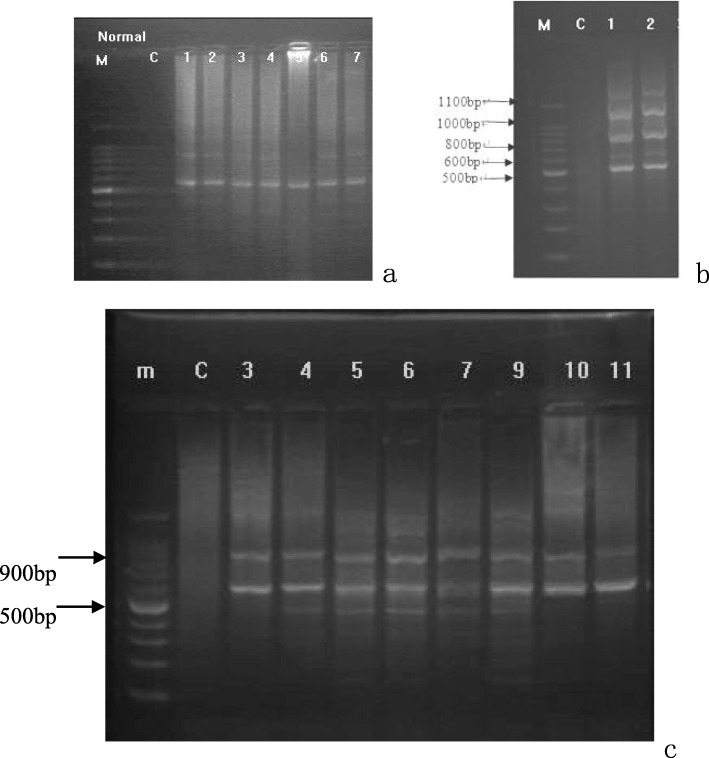
Electrophoresis pattern of RAPD results of *C. albicans* from the healthy group (2**a**: 1–7 electrophoresis lanes), E-OLP group (2**b**: 1–2 electrophoresis lanes), and NE-OLP group (2**c**: 3–11 electrophoresis lanes). M, Marker; C, control. (The numbers of strains did not relate to the numbers of cases)

Electrophoresis analysis of RAPD products showed that the healthy controls had only one band, both E-OLP isolates had multiple bands, and all the eight NE-OLP isolates had two bands (Fig. 2). S_AB_ analysis further showed that the NE-OLP formed a major clade, and the E-OLP formed another separate clade. The health control was basal to the NE-OLP and E-OLP clades.

**Fig. 3:**
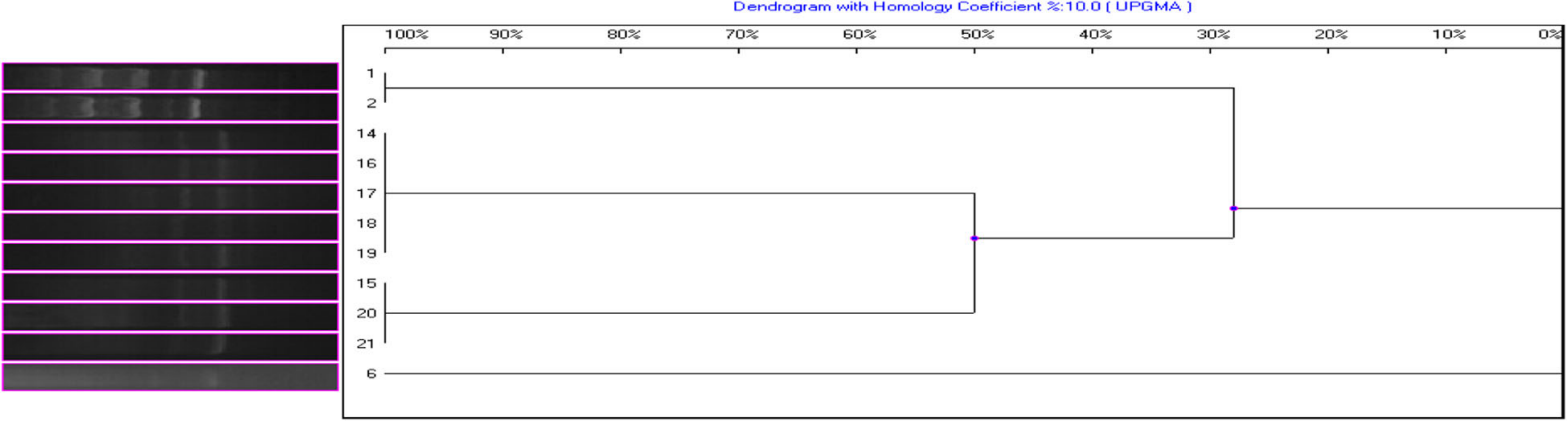
Genotyping tree of gene homology coefficient S_AB_ of *C. albicans* by UIV Band analysis. Horizontal axis = S_AB_; Vertical axis = genotypes of RAPD products of isolated strains . 1–2 = E-OLP strains no. **2, 8**, S_AB_ = 100%; 14, 16–19 = NE-OLP strains no. **3–7 and 11**, S_AB_ = 100%; 15, 20, 21 = NE-OLP strains no. **9, 10, and 12**, S_AB_ = 100%; 6 = No. **1** strain from the control group. (The numbers of strains did not relate to the numbers of cases).

Then, these RAPD genomes were analyzed by UIV Band. The results showed that genetic homology coefficient S_AB_ was less than 30% between the control, NE-OLP, and E-OLP groups, while it was 100% in a single subgroup. Additionally, the NE-group could be categorized into two subgroups, namely two genotypes, one including strains numbered 3–7 and 11, and the other including strains numbered 9, 10 and 12 (Fig. 3).

### ITS sequences

**Fig. 4:**
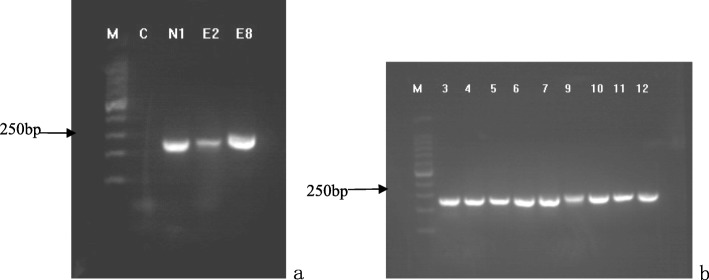
Electrophoresis patterns of ITS PCR results (all 250 bp) of strains. 4**a**: N1 strain from the control group, and E2 and E8 strains from the E-OLP group. 4**b**: 3–7 and 9–12 strains from the NE-OLP group. (The numbers of strains did not relate to the numbers of cases)

**Fig. 5:**
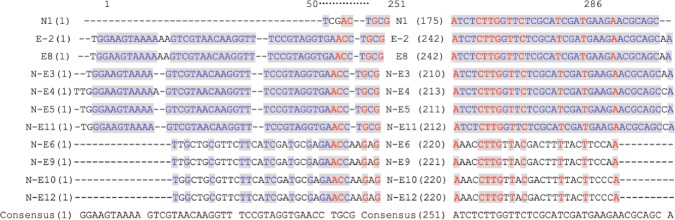
Sequences analysis results using Vector NTI Suite. 5′-TCGACTGC…AAGAACGCAGC-3′ was named as Type I for no. 1 strain from the control group of 101 subjects; 5′-GGAAGTAA…GAACGCAGCCA-3′ was named as Type IIa for strains no. 3–5, 7 and 11 from the NE-OLP group of 96 cases; 5′-TGGCTGCG…TTTACTTCCCA-3′ was named as Type IIb for strains no. 6, 9, 10 and 12 from the NE-OLP group of 96 cases; 5′-TGGAAGTA…GAACGCAGCAA-3′ was named as Type III for strains no. 2 and 8 from the E-OLP group of 53 cases. (The numbers of strains did not relate to the numbers of cases)

The DNA fragment with expected size (250 bp) was successfully amplified from all isolates (Fig. 4). BLAST analyses of the sequences referring to NCBI databases clearly showed that all isolates belonged to the *C. albicans* species. Then, these ITS sequences were genotyped using Vector NTI Suite software, and the sequence analysis showed that the base pair genotype results of ITS regions of the strains were consistent with the results of the RAPD analysis (Fig. 5). Specifically, the type I was found from normal strains, type IIa and IIb were detected from NE-OLP cases, and type III was identified from E-OLP cases.

## Discussion

The relationship between *C. albicans* and the etiology of OLP has long been puzzling many researchers and it still remains to be elucidated. The present study revealed that *C. albicans* from OLP were quantitatively and qualitatively distinctive with heathy individuals. Moreover, *C. albicans* isolates from both NE-OLP and E-OLP were genetically distinguishable from those in control group. These suggest that OLP might be a predisposing condition to *Candida* infection and certain specific genotypes of *C. albicans* are probably involved in occurrence and progression of OLP.

Generally, the prevalence of *C. albicans* in OLP patients differs from that found in healthy individuals. It has been reported that a positive *Candida* culture is more prevalent among OLP patients (48.9%) than among control subjects (26.7%) [13]. In the present study, we also identified a definitely higher positive frequency of *C. albicans* in the NE-OLP group and E-OLP group compared to healthy individuals. This discrepancy might cause the dysfunction of lymphocytes in OLP. As Simark-Mattsson et al showed the proliferation and cytokines production of peripheral blood mononuclear cells from OLP were significantly reduced following the stimulation of *C. albicans*, thus reflecting a potential immune regulatory mechanism of OLP modulating by *C. albicans* [9]. The high prevalence of specific *C. albicans* isolates in OLP suggest that they might potentially have strong adaptability to the microenvironment under the dynamic interaction between OLP progression and the fungal strains [21]. Yet, this is not consistent with results reported by Artico et al, who found that the positive prevalence of colonization by *Candida* spp. was higher in healthy subjects than in OLP [11]. The possible explanation for this discrepancy may lie in the differences of the sample size and methods including sample collection sites.

In the present study, the RAPD and ITS sequences were both chosen, because other studies failed to further classify *C. albicans* among different groups situation, with no better precision than telling just that they were *C. albicans*, and above reported two methods’ results had a clear coherence and consistency. In fact, it is a well-known fact that phenotyping is vulnerable to being influenced by environment and involved in poor repeatability, weak identification, and instability [22]. In contrast, genotyping improves the precision of *C. albicans* species and subtypes [23]. What supports the importance of genotyping is that *C. albicans* does not differ in phenotype between dimorphologicly respective symbiotic and pathogenic situations.

Although other technologies as multilocus sequence typing and microsatellite typing are thought to be more efficient and reliable, and even if the RAPD and ITS-PCR are viewed as obsolete by some scholars, we found them to be very useful. As a result, RAPD products showed by electrophoresis analysis that the healthy controls had only one band, E-OLP isolates had multiple bands, and all the eight NE-OLP isolates focused on two bands, and were further confirmed by ITS sequence. Moreover, the genetic homology coefficient S_AB_ was less than 30% between the control, NE-OLP, and E-OLP groups, while it was 100% inside subgroup. Previous genotyping results have usually revealed that *C. albicans* infection in OLP are exogenous [24], while authors here think that if they were exogenous, they should be disordered or similar in E-OLP or NE-OLP instead of the ordered genotypes. In addition, the exogenous colonization of *C. albicans* should reveal some indefiniteness and randomness. Nonetheless, a common natural symbionts *C. albicans* in OLP has not yet been found, which is why we are more inclined toward our initial hypothesis. On the contrary, we assumed that it was the endogenous genotypic changes of oral *C. albicans* under special environmental and ecological conditions that led to development of OLP. This might be because the specific microenvironment fostered a mutation of *C. albicans* from symbionts to further specific pathogenic genotypes which is also supported by other researches [15, 21, 22] and hopefully future molecular epidemiology, and to induce a T-cell-mediated immune response. As for elements of microenvironmental oral cavity including PH, temperature and ingredients in salivary or gum fluids, they have been identified to induce pathogen’s virulence key gene mutation and immune reaction or new balance [25]. Also, it is possible that the mutation drives *C. albicans* to get involve in and better adapt to a current environment [26], thus forming the type of dominant pathogen, which eventually affects the severity of the disease upon its interaction with the host. In this study, the results of both RAPD and ITS sequence showed that both intra-species homology and inter-species variations existed in *C. albicans* among the three clinical groups. The correlation between pathogen mutation and clinical progression of OLP was reflected in the changes from type I for normal strains, to type IIa, type IIb for NE-OLP, and to type III for E-OLP strains. Besides the underlying implication that an endogenous mutation of *Candida* might be the key to uncovering the etiology of OLP, these findings can potentially be used as indicator for the severity evaluation for OLP, as well as a therapeutic basis for individualized treatment.

**Fig. 6:**
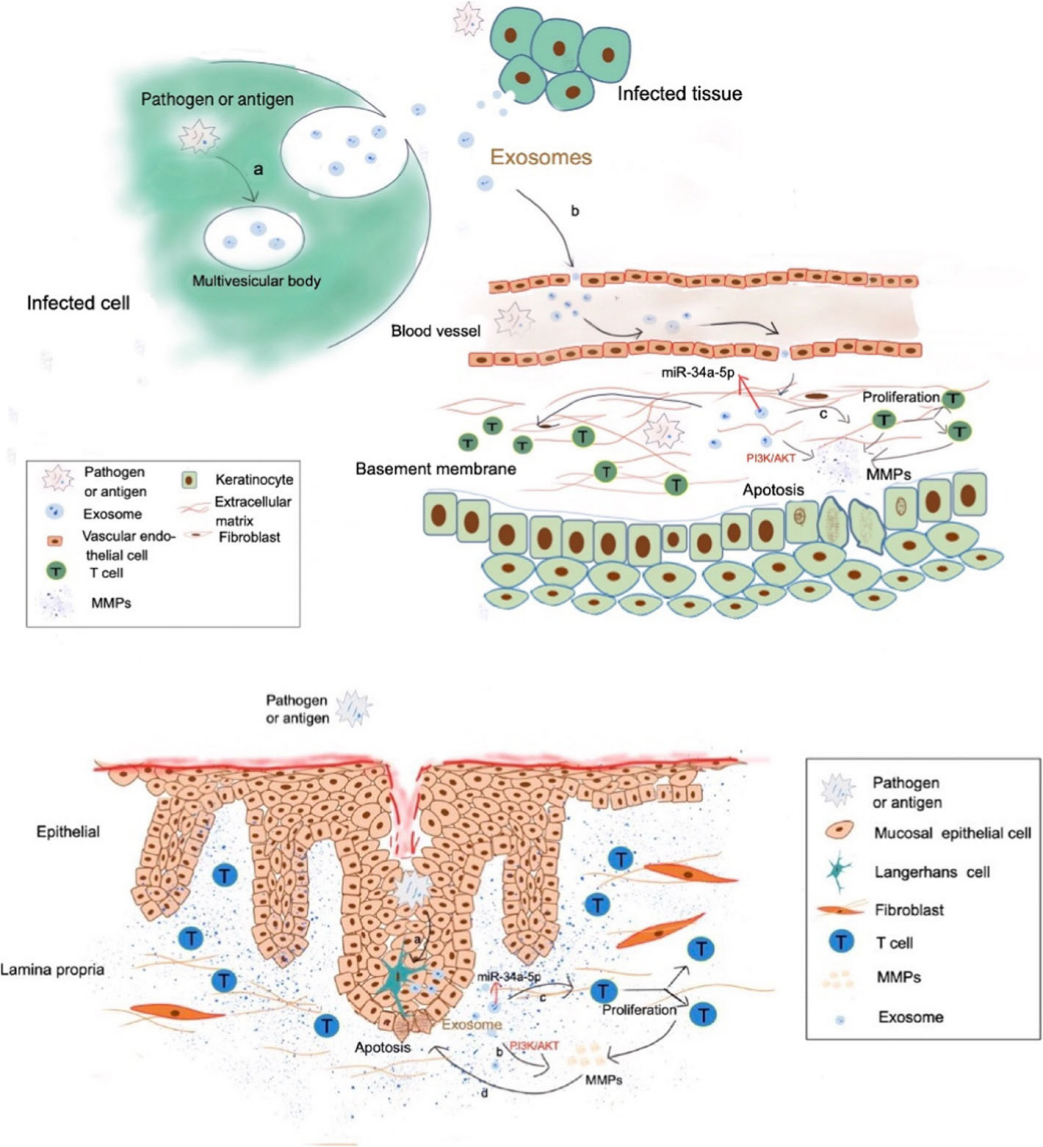
This figure further illustrates the possible pathogen etiology for OLP. The authors view fungi e.g. *Candida* pathogen/antigen as a vital part of the etiology and pathogenesis for OLP. This figure is the authors own as Hong He et al. illustrated in p 5316–5317 of 5313–5323 American Journal of Translational Research, 2019,11(9), about pathogen/antigen acts via a, b, c molecular affairs in possible pathogenesis of OLP. Also please kindly acknowledge there is also a small “a” among epithelia cells

Although previous studies have reported that OLP attracts *C. albicans*’ collection in lesions [15], we assume that *Candida* is probably the initial pathogen and the antigen for OLP (Fig. 6), since that OLP can be easily cured by addressing oral hygiene and dental health problems, which quite often involve fungi. If not so, solving the oral hygiene, dental and oral health, consuming antifungal drugs should have never worked. From these clinical aspects, the curative effect is comparatively easier in treating E-OLP than NE-OLP with superficial consumption of corticosteroids, and then subsequently or simultaneously addressing dental and hygiene problems same as treating NE-OLP. Correspondingly, **overlooked patients’ poor oral hygiene and health habits are indeed the cause for substantially prolonged healing and recurrences of OLP showing in our daily work and also mentioned in other studies**. In fact, practice experience tells us that **addressing oral hygiene and dental problems (which tend to involve numerous fungi and *Candida*) is a most effective way to cure OLP in clinical work with more accuracy than** other therapies such as adoption of immune drugs or herb products and similar medicine.

Accordingly, a strong positive relationship has also been identified between the *Candida* infection in oral cavity and the degree of epithelial dysplasia or OSCC [3, 27]. Gainza-Cirauqui and colleagues suggested that *C. albicans* isolated from potentially carcinogenic oral diseases could produce mutagenic amounts of acetaldehyde, which are involved in abnormal epithelial proliferation of mucosa [28]. This further induced adverse events like disorders and formation of strange cellular keratin that deteriorates the abnormal nuclear divisions and cell proliferation in epithelial plaque, thus developing to cancers [29]. Therefore, clinicians should pay special attention to the presence of *Candida* altering from combionts to pathogenic, and any premalignant dental-to-mucosa friction injury when treating patients with OLP and OLP with epithelial dysplasia or carcinoma.

Our results with the clinical *C. albicans* strains confirmed that the ITS sequences and RAPD homology coefficient S_AB_ of *C. albicans* were obviously different among E-OLP, NE-OLP and healthy individuals, thus suggesting that endogenous *C. albicans* in oral environment may be involved in the etiology and pathogenesis of OLP. Although the sample of strains in this study was small, and the mutual effect between OLP lesion and *C. albicans* gene mutation is dynamic and complex, reported results are strong. Nonetheless, future studies with larger sample size or an OLP animal model are required to further verify reported findings.

## Conclusions

The etiology of OLP might be explained by the endogenous infection of *Candida* and its gene mutation under specific and dynamic microenvironment of the patient’s oral cavity. These results should be further used to confirm the real etiology, as well as more precise and effective therapeutic strategies for OLP. This is crucial for correct intervention of premalignant progression in oral health.

## Data Availability

The authors would deliver their raw data to him who any reader contacts asking for raw data.

## Abbreviations

API: Analytical Profile Index
BLAST: Basic Local Alignment Search Tool
*C. albicans*: *Candida albicans*
DNA: DeoxyriboNucleic Acid
E-: Erosive-
IRB: Institutional review boards
ITS: Internal transcribed spacer
NE-: Non-erosive-
OLP: Oral lichen planus
OSCC: Oral squamous cell carcinoma
PCR: Polymerase chain reaction
RAPD: Random amplified polymorphic DNA
